# Imaging Clinical Subtypes and Associated Brain Networks in Alzheimer’s Disease

**DOI:** 10.3390/brainsci12020146

**Published:** 2022-01-23

**Authors:** Karl Herholz

**Affiliations:** 1 Division of Experimental Psychology and Neuroscience, University of Manchester, Manchester M139PL, UK; karl.herholz@manchester.ac.uk; 2 Sheffield Institute for Translational Neuroscience, University of Sheffield, Sheffield S102HQ, UK

**Keywords:** Alzheimer’s disease, early diagnosis, posterior cortical atrophy, progressive aphasia, positron emission tomography, magnetic resonance imaging

## Abstract

Alzheimer’s disease (AD) does not present uniform symptoms or a uniform rate of progression in all cases. The classification of subtypes can be based on clinical symptoms or patterns of pathological brain alterations. Imaging techniques may allow for the identification of AD subtypes and their differentiation from other neurodegenerative diseases already at an early stage. In this review, the strengths and weaknesses of current clinical imaging methods are described. These include positron emission tomography (PET) to image cerebral glucose metabolism and pathological amyloid or tau deposits. Magnetic resonance imaging (MRI) is more widely available than PET. It provides information on structural or functional changes in brain networks and their relation to AD subtypes. Amyloid PET provides a very early marker of AD but does not distinguish between AD subtypes. Regional patterns of pathology related to AD subtypes are observed with tau and glucose PET, and eventually as atrophy patterns on MRI. Structural and functional network changes occur early in AD but have not yet provided diagnostic specificity.

## 1. Introduction

Dementia is the result of vast brain damage that has been accumulating over a long time, typically many years, probably even decades. These changes are largely irreversible once dementia has set in. Thus, any treatment to prevent dementia would need to start before dementia onset, and early diagnosis is therefore paramount [1]. However, early-stage symptoms may be very mild and quite diverse among patients. Symptoms tend to cluster into different clinical syndromes which characterise subtypes, and there are also indications that these subtypes might be associated with different pathophysiological processes [2]. This likely means that early-stage treatment would need to be adjusted to the respective subtype and associated pathophysiology. Therefore, tools for early and specific diagnosis of subtypes are required. This review will describe the suitability of imaging methods to provide diagnostic information on subtypes at an early stage.

The review is based primarily on a PubMed and Google Scholar searches with keywords “Alzheimer disease”, “subtypes”, “posterior cortical atrophy”, “logopenic aphasia” in combination with “positron emission tomography”, “magnetic resonance imaging” or “network”. Emphasis was given to most recent and key original publications.

## 2. Alzheimer’s Disease Subtypes

For many years, it has been noted that Alzheimer’s disease (AD) does not present a unitary pattern of symptoms in all patients [3]. While a broad range of cognitive and neuropsychiatric symptoms may accompany the manifestation of AD [4], a number of distinct clinical subtypes have been identified. Amyloid, tau, and neurodegeneration biomarkers, according to the A/T/N scheme [5], are important for early diagnosis of AD. Among those, imaging biomarkers are most frequently used to investigate AD subtypes.

Most prominently, the clinical syndromes of logopenic progressive aphasia (LPA) [6], posterior cortical atrophy (PCA) [7], and a rare frontal variant of AD [8] have been identified as “atypical presentations” [9] in contrast to the most frequent presentation of AD with leading episodic memory impairment. The leading symptoms of these atypical presentations have been reviewed in detail by [10]. They are related to neocortical focal dysfunction and include visual dysfunction, such as simultagnosia, and constructional dyspraxia in PCA, aphasia with impaired word retrieval and repetition in LPA, and executive or behavioural dysfunction in frontal variant AD. Initially, they show relatively little memory deficits related to hippocampal dysfunction. It has been noted that differences between syndromes are most distinct at the early stages of AD and become more homogeneous as the disease progresses to severe dementia [3].

Recent diagnostic research criteria [5] define AD as a biological disease which ultimately leads to dementia, but at an early stage may be present already in subjects with normal cognition or mild cognitive impairment (MCI). It is characterised by pathological deposits of beta-amyloid and tau as markers that are common to all subtypes. While the distribution of amyloid is rather similar in all subtypes, there are marked differences in the distribution of tau between subtypes [11]. Imaging studies have led to the classification of AD subtypes as either typical, limbic-predominant, or hippocampal-sparing [12]. The hippocampal-sparing subtype is often associated with “atypical” clinical presentations [13], especially LPA and PCA, early dementia onset and rapid progression. This is therefore the main focus of this review. In contrast, the limbic-predominant form with leading memory deficits is often associated with old age and progresses more slowly.

Rare monogenetic forms of AD due to APP, PS1 or PS2 gene changes may present leading memory deficits but also otherwise uncommon focal neurology signs such as myoclonus or seizures [14]. They are not considered further in this review as they are linked to rare family pedigrees and do not play a significant role in normal clinical settings.

There is good correspondence between the regional distribution of tau deposits and clinical symptoms [11]. One may therefore hypothesise that biological factors determining regional tau distribution may also be main determinants of clinical presentation. Such factors of regional vulnerability might be due to other previous or coexisting diseases or genetic predispositions without direct relation to Alzheimer’s disease [15,16]. Another important mechanism for preservation of some cognitive functions at an early stage may be the compensation of regional dysfunction by remaining intact brain networks [3].

One cannot exclude the possibility that AD subtypes may be caused different etiological mechanisms. There is no direct evidence for that, but atypical presentations are more frequently observed in patients with early onset of dementia (before age 65), are less frequently associated with the apolipoprotein E4 genotype, often progress more rapidly than typical AD with leading memory deficits in old age, and there may also be molecular differences in amyloid and tau pathophysiology [17]. Thus, the efficacy of emerging disease-modifying therapies targeting specific pathophysiological mechanisms in AD might also differ between subtypes.

The clinical presentation of atypical AD can be similar to non-AD diseases, such as fronto-temporal dementia (FTD) or Dementia with Lewy bodies (DLB). These diseases show distinct characteristics on pathological examination, although there is possible overlap of tau and deposits of another common pathological protein, TAR DNA-binding protein 43 (TDP-43), between AD and FTD [18], while amyloid deposits are also commonly found in DLB. Vascular pathology may make a significant contribution to cognitive impairment and brain damage in Alzheimer’s disease and thus modify clinical presentation and accelerate progression [19]. Therefore, differential diagnosis between the various subtypes of AD and non-AD diseases at an early stage poses a significant challenge.

While AD subtypes have originally been defined by clinical and pathological characteristics, the ever-increasing scope of imaging methods and the application of machine-learning approaches to classification of imaging patterns [20] have led to multiple alternative subtype classifications [21]. Image data-driven analyses identified limbic-predominant and cortical-predominant subtypes, the latter roughly corresponding with atypical clinical presentations. At the prodromal stage of mild cognitive impairment (MCI) this roughly corresponds to amnestic and non-amnestic subtypes [22]. Ultimately, a large variety of variables, including neuropsychology, pathology, genetics, proteomics, as well as structural, functional, and molecular imaging are to be considered for distinction between AD subtypes. Criteria for defining subtypes vary between studies. While there is broad overlap between imaging-defined hippocampal sparing and clinically or pathologically defined atypical presentation subtypes, only few studies use both types of criteria [12,13,23].

## 3. Imaging Techniques

### 3.1. Amyloid PET

PET imaging of fibrillary plaques containing beta amyloid provides the most direct technique for distinguishing between AD and non-AD diseases. Three 18F-fluorine labelled PET tracers, florbetapir [24], florbetaben [25] and flutemetamol [26], have been developed and validated by comparison with post-mortem pathology for clinical use. Their sensitivity for detection of fibrillary amyloid plaques is 90% or better. Therefore, a negative amyloid PET scan is a reliable indicator for exclusion of AD in patients with mild cognitive impairment or dementia. Only in extremely rare Nordic genetic mutations pathological fibrillary amyloid plaques have been found in subjects who had negative amyloid PET scans [27].

The role of amyloid PET for clinical diagnosis of Alzheimer’s disease is a complex issue. This is mainly due to possible amyloid co-pathology in patients with other neurodegenerative diseases [28] and age-related amyloid deposits in cognitively normal old subjects without clinical significance. Therefore, a probabilistic approach to diagnosis, also considering the clinical presentation and age, has been suggested [29].

Consistent with pathoanatomical data [30], logopenic progressive aphasia shows amyloid positivity on PET scans more frequently than other types of progressive aphasia [31,32]. As not all forms of progressive aphasia are easily classified neuropsychologically, and some cases of non-fluent progressive aphasia may also show amyloid pathology [33], amyloid PET is a useful diagnostic tool to determine etiology in clinical cases of progressive aphasia [34].

AD subtypes show a similar regional distribution of amyloid plaques [35,36,37], with only minor left-right asymmetry in LPA [38] or small anterior-posterior shift in PCA [39]. Therefore, the standard clinical implementation of amyloid PET as a static scan does not provide a differentiation between AD subtypes. It also does not provide evidence of functional impairment, which is important for assessing the clinical stage of the disease. However, in combination with markers of functional neuronal impairment, amyloid PET is a powerful tool to predict progression to dementia in patients with mild cognitive impairment [40]. A combination of both markers can be obtained by dynamic amyloid PET in a single scanning session [41,42]. Early tracer uptake immediately after injection reflects cerebral blood flow as a marker for functional impairment, and is also correlated with FDG [43]. Therefore, it may also provide some differentiation between AD subtypes similar to FDG (see Section 3.3), but this is still to be demonstrated.

### 3.2. Tau PET

Pathological deposits of tau in neurofibrillary tangles are another hallmark of AD. However, pathological tau deposits may also occur in other neurodegenerative diseases, often with distinct molecular and microstructural differences. Tau PET tracers have been mainly developed for neurofibrillary tangles in Alzheimer’s disease, which contain 3- and 4-repeat isoforms of tau [44]. There is also some off-target binding to structures, such as the choroid plexus, that do not contain neurofibrillary tangles. It has been demonstrated that the most widely used tau tracer 18F-flortaucipir (previously known as 18F-AV1451) reflects high pathological Braak stages (stages V and VI) [45]. It does not detect earlier Braak stages reliably and does not bind strongly to tau isoforms in progressive supranuclear palsy and other non-AD tauopathies [46]. In spite of these reservations, it may have a potential for differentiation between AD and non-AD diseases similar to amyloid PET [47,48].

Tau PET has been developed approximately ten years after amyloid PET and therefore evidence on clinical use is still limited. Second-generation tau PET tracers have recently been introduced [49,50,51] and more are still under development [52]. Recent comparisons show that monoamine oxidase B-related off-target binding, which has been observed with 18F-flortaucipir in striatum and chorioid plexus, is reduced or absent in second generation tracers [51], while binding to cortical tau inclusions composed of paired helical filaments is similar [53]. 18F-MK6240 demonstrated improved dynamic range [54] which may make assessment of earlier Braak stages possible, and some off-target binding in meninges. Similar observations were reported for 18F-RO948 [55] and 18F-PI2620 [56].

In contrast to amyloid, the localisation of tau deposits correlates with clinical symptoms. Therefore, Alzheimer’s disease subtypes can be characterised by tau PET. In cross-sectional studies [57,58], patients with predominant memory impairment showed an increased uptake of 18F-flortaucipir in medial temporal lobes, those with predominant visuospatial dysfunction (as in PCA) increased occipital and right temporoparietal uptake, and language impairment (as in LPA) was predominantly associated with left-sided temporoparietal uptake. It has also been possible to reproduce the three pathoanatomical fibrillary tangle subtypes (hippocampal sparing, limbic predominant, and typical with balanced tangles counts in hippocampus and association cortices) with 18F-flortaucipir PET in AD patients at estimated Braak V-VI stage [59].

Age is an important factor, with a generally higher uptake in younger patients [60] and an increased ratio of medial temporal to neocortical uptake in older patients and patients with ApoE4 genotype [61]. Findings were confirmed in longitudinal studies which also demonstrated that both clinical and tau progression rates were more rapid in atypical and early onset cases [62].

In PCA, 18F-flortaucipir was selectively retained in posterior brain regions that were affected clinically and also showed reduced FDG uptake [63], while patients with LPA typically show left greater than right temporo-parietal tracer uptake [57]. Subtype-related regional patterns are generally stable during progression from MCI to Alzheimer dementia [64], while all subtypes share an increase in frontal tracer uptake during progression of AD [65].

Tau PET patterns discriminate between logopenic and other types of progressive aphasia [66], but increased binding of 18F-flortaucipir was also found in the anterior temporal lobe of semantic aphasia patients, who would typically have underlying TDP-43 pathology rather than a tauopathy [67,68]. The situation may improve with introduction of second-generation tau tracers [49] and further research into the molecular binding mechanisms of these tracers.

Data-driven analysis of 18F-flortaucipir PET [69] in patients with Alzheimer dementia revealed three clusters: one with low entorhinal and cortical uptake, one with high cortical and entorhinal uptake, and one with low entorhinal but high cortical uptake. Patients with atypical clinical presentation were mostly included in the latter cluster, while balanced high or low uptake was associated with typical presentation. A recent comprehensive longitudinal multicenter tau PET study involving multiple tau tracers and all clinical stages of AD [70] identified four subtypes of AD: limbic, hippocampus-sparing, occipital, and temporo-lateral. These subtypes were largely stable during follow-up and did not just represent different disease stages during progression. Occipital and (predominantly left) temporo-lateral subtypes were loosely related to clinical subtypes PCA and LPA, respectively, but also involved mesial temporal tau deposits. Clinical progression was most rapid in the temporo-lateral subtype.

In addition, tau PET studies have contributed to exploration of a “tau-first” subtype of AD, which was found to represent up to 38% of cases in the ADNI cohort [71]. In these cases, tau deposits are found in mesial temporal lobe structures prior to evidence of amyloid deposits. As also observed in another study [72], in presence of fibrillary amyloid, tau then also spreads to neocortical structures and patients develop dementia. Tau-first patients tend to be younger than patients with the classical amyloid-first sequence and show signs of increased microglial activation [71].

Several longitudinal tau PET studies [73,74,75,76,77] provided in-vivo evidence for the spread of tau through structural and functional networks, as hypothesised earlier based on experimental and pathological studies [78]. This may involve anterograde (Braak stage-like) and retrograde propagation [79]. Specific networks will be addressed in subsequent sections on MRI imaging of networks.

### 3.3. FDG PET

**Figure 1:**
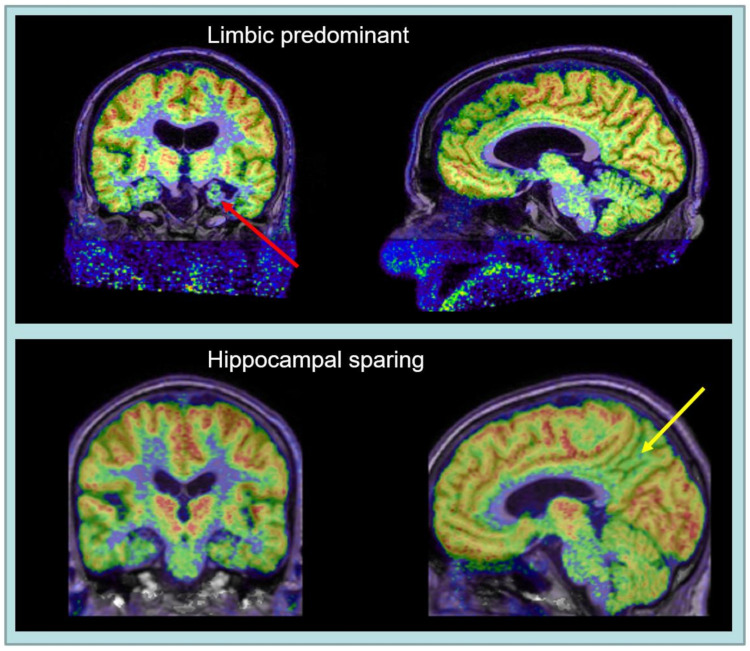
FDG PET with fusion overlay on T1-weighted MR, demostrating hippocampal hypometabolism and atrophy (red arrow) in limbic-predominant AD (**top**) and impaired parietal hypometabolism (yellow arrow) in a hippocampal-sparing AD (**bottom**). Both cases show metabolic impairment of posterior cingulate cortex.

Based on the coupling between regional cerebral glucose consumption and neuronal function, PET with 18F-2-deoxy-2-fluoro-D-glucose (FDG) has been an excellent method for biomedical and functional characterisation of AD subtypes (Figure 1) over many years, as reviewed by [4]. In particular, neocortical metabolic deficits corresponding to early cognitive impairment and neuropsychiatric symptoms in atypical presentations of Alzheimer’s disease have been demonstrated by FDG PET with high sensitivity [80,81]. Moreover, working memory problems, often prominent in early onset AD, have been associated with the corresponding neocortical network [82]. Metabolic impairment has also been observed in hippocampus [83], but not as consistently as neocortical metabolic impairment and as expected due to hippocampal atrophy [84].

Posterior cortical atrophy (PCA) is characterised by metabolic impairment, predominantly in occipital and occipito-temporal association cortices [85] already at an early stage [36]. The temporo-parietal metabolic deficits of Alzheimer’s disease are also present. The pattern is similar to dementia with Lewy bodies (DLB) but there are some noticeable differences. DLB is associated with metabolic impairment in primary visual cortex which usually is spared in PCA, while the posterior cingulate gyrus is relatively preserved in PCA (“cingulate island sign”) [86]. However, some studies found a considerable overlap of patterns [87] or better discriminatory power for PCA with metabolic reduction in lateral temporal than in posterior cingulate cortex [88].

Logopenic progressive aphasia (LPA) is associated with predominantly left-sided temporo-parietal hypometabolism [89]. However, progressive aphasia syndromes including LPA can also be caused by other pathologies [18]. As mentioned, distinction is possible by amyloid PET. Amyloid negative LPA patients tend to show a higher involvement of frontal and anterior temporal areas [90,91].

The rare frontal variant of AD (fvAD) has been characterised by dense neurofibrillary tangles in the frontal lobe and leading impairment of behaviour or executive function [8]. With FDG PET frontal lobe hypometabolism has been observed including orbitofrontal and mesial frontal cortices, which are usually spared in typical AD [92,93]. Frontal and parietal regions overlapping with working memory networks are also impaired frequently in fvAD [94] while temporal poles are frequently affected in FTD. In group-wise comparison, fronto-mesial metabolic impairment is more severe in FTD than in AD and even in fvAD some temporoparietal metabolic impairment is still present, but differences are probably not strong enough to allow individual diagnostic distinction. Widespread and diffuse frontal metabolic impairment is common in many conditions including but not limited to depression and vascular cognitive impairment, and there is also a significant frontal age effect [95]. Obviously, amyloid PET could provide a clearer distinction between AD and frontotemporal dementia [96], but still there is a possibility of co-pathology that may complicate diagnostic classification [28].

In a data-driven analysis of amyloid-positive AD patients from the ADNI cohort three main hypometabolic subtypes were identified. The vast majority showed the classic posterior temporo-parietal hypometabolic pattern with or without prominent hippocampal hypometabolism, the latter probably including cases of PCA and LPA. A small subgroup showed predominant frontal cortex hypometabolism with executive dysfunction [97]. At the MCI stage an additional cluster without significant hypometabolism and little progression of cognitive impairment was identified, confirming previous studies about the prognostic value of FDG PET in MCI.

In normal controls, metabolic activity shows regular regional correlation patterns which partially correspond to functional networks identified with fMRI [98]. A loss of regional correlations was observed in typical AD, even in cases with moderate reduction in regional metabolic activity, while some regional correlations were preserved in atypical AD [99].

Cerebral glucose metabolism and cerebral blood flow (CBF) are correlated in normal controls and patients with neurodegenerative diseases [100,101]. Therefore, CBF imaging techniques, which include 15O-water PET [102], single photon emission computed tomography (SPECT) with 99mTc-HMPAO [103] and similar tracers, and arterial spin-labelling MRI [104] have also been suggested as diagnostic and research methods in AD. While generally good correspondence has been observed, there were also some regional differences, mainly found in the hippocampus and frontal lobes [105,106]. As CBF values also depend on systemic parameters such as arterial pCO_2_, FDG PET tended to be more robust and more reliable in some direct diagnostic comparisons [107,108,109].

### 3.4. Structural MRI

Even without quantitative analysis structural T1-weighted MRI can provide visual scores of hippocampal atrophy, which is most prominent in limbic predominant and typical subtypes of Alzheimer’s disease. Modern quantitative image processing techniques allow analysis of the regional distribution of atrophy across the entire brain, demonstrating characteristic patterns in neurodegenerative diseases [110] and also showing differences between parietal-predominant, medial temporal-predominant, and diffuse atrophy AD subtypes [111].

**Figure 2:**
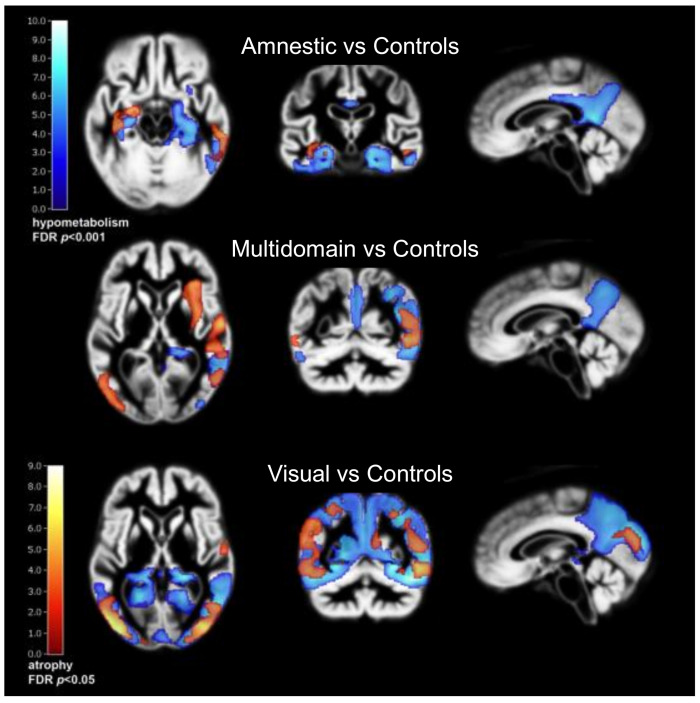
Metabolic impairment on FDG PET (blue) and corresponding atrophy (red), in similar location but less extensive, in three subgroups of AD which have been described in [99]. C/f C. Kobylecki, University of Manchester, UK.

Regional atrophy tends to follow metabolic impairment on FDG PET (Figure 2), which is more sensitive than atrophy to detect prognostically important impairment of temporoparietal association cortex at an early stage [112]. Ultimately, atrophy patterns are similar to patterns of metabolic impairment observed with FDG PET in AD subtypes. AD patients with a temporoparietal-dominant and hippocampal-sparing atrophy pattern show more AD-like hypometabolism on FDG and faster subsequent clinical decline than patients with a limbic-predominant pattern [113,114]. This mainly applies to early-onset AD, while in non-demented subjects above age 70, typical hippocampal-predominant atrophy is most closely associated with subsequent progression to Alzheimer dementia [115].

The availability of structural MRI scans in large prospective cohorts enables data-driven studies of Alzheimer subtypes [20]. In addition to classical subtypes, they identified diffuse and minimal atrophy subtypes [116]. Most studies identify three of four subtypes [117,118] in patients across all clinical stages of AD, of which hippocampal plus parietotemporal atrophy carries the highest risk of progression to dementia, while hippocampal-only atrophy is thought to represent an earlier stage with slower progression. Atrophy patterns are also correlated with tangle density distribution [23] and PET tau imaging [119], but there are also interesting deviations. Das et al. [120] identified patient groups with more atrophy and more rapid progression than expected based on their tau PET scans and, in contrast, also groups that appeared relatively resilient to tau.

The Subtype and Stage Inference (SusStaIn) model addresses cross-sectional heterogeneity and temporal stages of neurodegenerative diseases [121]. When applied to MCI, subtypes were identified based on different patterns of initial ventricle enlargement with moderate predictive power (ROC AUC 0.76) for progression of cognitive impairment [122]. Complex models are being developed to describe longitudinal trajectories of AD subtypes [123], and an international competition (TADPOLE) has been conducted to find the best predictive models [124]. Advanced models also include additional biomarkers and cognitive parameters for prediction of progression [125].

Atrophy patterns can be related to changes in structural networks assessed by diffusion tensor imaging (DTI) with MRI [126]. Multimodal serial PET and MR scanning of cognitively healthy older individuals from the Harvard Aging Brain Study [127] demonstrated that increased mean diffusivity of the hippocampal cingulum bundle predicted tau accumulation in downstream-connected posterior cingulate cortex and memory decline in amyloid-positive but not in amyloid-negative individuals, supporting the concept that structural network changes guide amyloid-dependent extrahippocampal spread of tau. Ultimately, reductions in hippocampal connectivity correlate with hippocampal tau in MCI and AD, and with amyloid in the target regions of those connections [128].

MRI also is an important tool for assessing vascular brain damage [129]. While vascular dementia usually is the consequence of ischemic infarcts or haemorrhage that affect functionally important brain areas, vascular cognitive impairment is a common condition which may also contribute to neurodegenerative processes and AD especially in old patients [130,131]. Lesions in white matter are detected with T2-weighted MRI with high sensitivity, and with DTI such lesions and loss of fibre integrity can be localised to specific neuronal tracts. These tracts form the structural connectome of the brain [132], which can be linked with correlated functional and structural cortical changes for a comprehensive assessment of brain networks in normal aging and dementia [126].

In PCA, the combination of DTI with FDG PET demonstrated the degeneration of the major anterior-posterior fibre bundles and commissural frontal lobe tracts. These were associated with hypometabolism not only in posterior temporal, parietal, and occipital cortex, but also in the frontal eye fields [133]. The pattern is different from typical AD, and even in a longitudinal study hippocampal, entorhinal and frontal regions underwent a low rate of change in PCA and never approached the extent of posterior cortical involvement [134].

Similar to hypometabolism in FDG PET, LPA is primarily associated with left temporoparietal atrophy and impairment of a dorsal language network that causes word finding problems and phonological errors [135,136].

### 3.5. Functional MRI and Network Analysis

Functional MRI (fMRI) has become a standard technique for analysis of correlations between regional activities and for defining functional brain networks. The most prominent and relevant is the default mode network (DMN), connecting the posterior cingulate cortex as a central hub with prefrontal and medial temporal cortices [137]. It is highly active in controls during cognitive rest, while other networks become more active during cognitive or sensory motor stimulation. Functional networks in AD are mostly studied at a cognitive resting state because functional activation fMRI depends on task performance which varies in AD and therefore is a potential confounder.

Accumulation of amyloid is associated with changes in the topology of structural connectivity [138] and of functional DMN connectivity already at preclinical stages of AD [139]. A study based on longitudinal data suggested failure of the functional posterior DMN connectivity even before accumulation of amyloid, followed by a shift in functional connectivity to frontal lobe hubs while amyloid is accumulating [140]. In a combined FDG PET and fMRI study, impairment of directional signalling within the DMN was associated with cognitive deficits in patients more strongly than any of regional PET and fMRI measures [141]. However, such network alterations are not specific for AD but also occur in a large variety of neurodegenerative and psychiatric diseases [142]. While functional brain connectivity depends on brain development and probably also education, it may also contribute to cognitive reserve and resilience in AD [143].

In the Swedish Biofinder Study, researchers found that PET tau distribution in AD primarily overlapped with the dorsal attention network, and also with higher visual, limbic and parts of the default-mode functional networks [144]. In another study, the intensity of tau deposits was highest in nodes with strong local functional connectivity [76]. In a data-driven comparison of functional and tau pathology networks in typical AD, a fair-to-moderate overlap was observed with known resting-state networks, particularly the DMN [145].

In PCA, fMRI demonstrated disruption of a dorsal attention network related to impairment of attentional circuits and memory performance [146] as well as reduced connectivity within the visual network and between the visual areas and frontal eye fields [147]. As to be expected, with disease progression functional and structural connectivity worsens from occipital to temporo-parietal and frontostriatal regions [148]. In contrast to typical AD, functional impairment in PCA does not affect the DMN, while dorsal and ventral visual networks may be affected differentially [149].

In LPA, reduced connectivity in the left temporal language network and parts of the left working memory network was observed when compared to typical AD, while there was less impairment of the DMN than in typical AD [150]. Impaired networks also differed from other progressive aphasia types, with semantic progressive aphasia showing also reduced connectivity with the anterior temporal lobe, and agrammatic progressive aphasia involving the inferior frontal gyrus [151]. A graph-theoretic analysis noted the appearance of new functional hubs in the right hemisphere [152].

**Figure 3:**
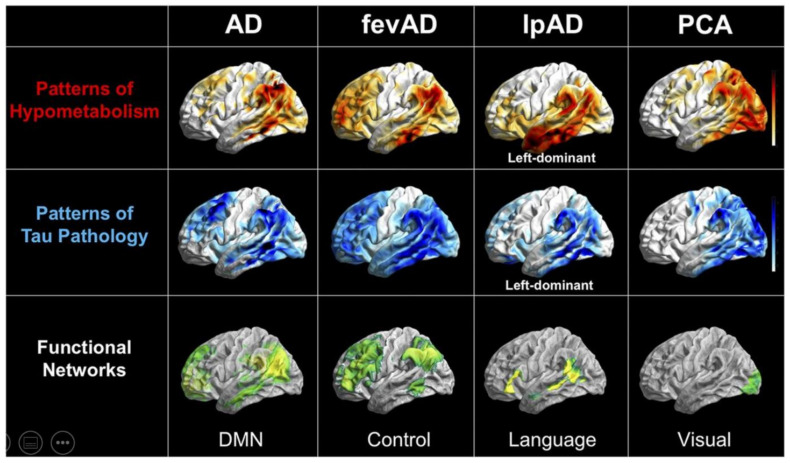
Conceptual depiction of a partial correspondence between hypometabolism (FDG PET), tau PET and functional networks in typical AD (**left**) and three subtypes, frontal-executive variant AD (fevAD), logopenic progressive aphasia (lpAD) and PCA. This figure was originally published in JNM [153]. Reproduced with permission and courtesy of Merle Hoenig, University of Cologne, Germany.

Associations between AD subtypes and functional networks are illustrated conceptually in Figure 3, while further research into pathophysiological mechanisms and exact funtional-anatomical relations is required.

### 3.6. Neurotransmitter and Neuroinflammation Imaging

PET and SPECT also offer possibilities for imaging of specific neurotransmitter systems. The nigro-striatal dopamine system received most attention, as it is impaired in dementia with Lewy bodies (DLB) but preserved in AD. Thus, imaging of dopamine synthesis [154] or D2 receptors [155] in the basal ganglia provides a diagnostic tool for differentiation between DLB (reduced activity) and PCA (normal activity) but does not differentiate between AD subtypes.

PET research studies are also conducted on the cholinergic system which is impaired in AD and other neurodegenerative disorders [156,157]. While cholinergic deficits correlate with memory function and are an important area of research, there is currently no evidence that they would contribute to a differentiation of AD subtypes [158].

A large range of other targets, including noradrenergic [159], mitochondrial [160] and synaptic vesicular markers [161] have been explored in AD. In recent years, imaging of activated microglia and astrocytes has attracted most interest as a research area with therapeutic potential [162,163]. It will likely be relevant for predicting disease progression at the MCI stage but so far has not been associated with specific AD subtypes.

## 4. Summary

**Figure 4:**
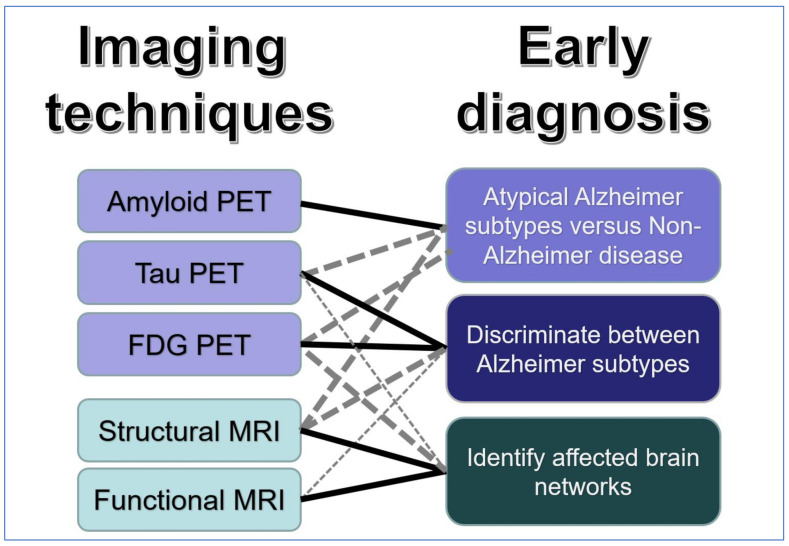
Graphical summary of main findings.

As illustrated in Figure 4, tau PET demonstrates characteristic regional patterns of tau deposits along known structural and functional networks which are associated with AD subtypes. These patterns probably provide good distinction between AD and non-AD types of dementia, but further research characterising different tau tracers and their ability to assess early clinical stages of AD is required.

The most direct and accurate distinction between AD and other types of dementia is provided by amyloid PET already at an early MCI stage. This is most useful with atypical clinical presentations. Amyloid PET can be used as an imaging biomarker in research, treatment and prevention trials even before onset of clinical symptoms, but it does not differentiate between AD subtypes.

FDG PET, as well as structural and functional MRI, distinguish between AD subtypes on the basis of the neocortical regions and networks involved. FDG PET provides high sensitivity to identify subtypes with atypical clinical presentations and risk of rapid progression already at the MCI stage.

MRI is widely available and large data bases allow application of AI techniques, such as deep learning, for identification of AD subtypes. Atrophy patterns are a relatively late marker of neurodegeneration which can be used to classify AD subtypes in symptomatic patients. Functional changes occur early and provide insight into the impairment of brain networks, but do not provide high individual diagnostic specificity.

## Data Availability

Not applicable.
